# Supplementary material for: Development of a patient-centred tool for use in total hip arthroplasty
Source: PLoS One. 2024 Oct 24;19(10):e0307752. doi: 10.1371/journal.pone.0307752 (PMC11500863; doi:10.1371/journal.pone.0307752)
Supplement: S1 File — (PDF) [file pone.0307752.s001.pdf]

Bénéfices et risques de la prothèse totale de hanche - Questionnaire médecin

1. Concernant la pose d'une prothèse de la hanche, les **<u>bénéfices</u>** suivants peuvent avoir une importance plus ou moins prépondérante, selon la situation de chaque patient. <u>D'une façon générale</u>, en moyenne, quelle est l'importance de ces bénéfices pour les patients ?

Pour chaque ligne ci-dessous, veuillez entourer le chiffre approprié :

		Insignifiant 1	2	3	4	Prépondérant 5
a.	Disparition de la douleur	1	2	3	4	5
b.	Arrêt ou diminution des médicaments contre la douleur	1	2	3	4	5
c.	Retrouver le sommeil	1	2	3	4	5
d.	Bien-être émotionnel	1	2	3	4	5
e.	Indépendance dans la marche et les déplacements	1	2	3	4	5
f.	Reprise des activités quotidiennes à la maison	1	2	3	4	5
g.	Reprise des activités professionnelles	1	2	3	4	5
h.	Reprise des activités de loisirs (sport, voyages, etc.)	1	2	3	4	5
i.	Reprise de sa vie sociale (voir ses amis, voir sa famille)	1	2	3	4	5

j. Autre(s) bénéfice(s), veuillez préciser : ....

....

....

Parmi la liste ci-dessus (a à j), merci d'indiquer les trois bénéfices <u>les plus importants</u> à vos yeux, par ordre d'importance :
(Merci de mentionner la lettre correspondante, par exemple « a » pour la disparition de la douleur, etc.)

1. ___________ 2. ______________ 3. ______________

2. Bénéficier d'une prothèse de hanche est synonyme de retrouver l'autonomie, l'indépendance, de récupérer la pleine possession de ses moyens. Dans quelle mesure êtes-vous d'accord avec cette opinion ?

Merci d'entourer le chiffre approprié ci-dessous

Pas du tout d'accord				Tout à fait d'accord
1	2	3	4	5

3. Concernant la pose d'une prothèse de la hanche, les **<u>conséquences indésirables ou les problèmes</u>** suivants peuvent avoir une importance plus ou moins prépondérante, selon la situation de chaque patient. <u>D'une façon générale</u>, en moyenne, quelle est l'importance de ces problèmes pour les patients

Pour chaque ligne ci-dessous, veuillez entourer le chiffre approprié :

		Insignifiant				Prépondérant
a.	Saignement important	1	2	3	4	5
b.	Caillot de sang (thrombose veineuse profonde)	1	2	3	4	5
c.	Décès lors de l'opération	1	2	3	4	5
d.	Douleur persistante	1	2	3	4	5
e.	Douleur qui se répand sur d'autres articulations	1	2	3	4	5
f.	Un bruit persistant de la prothèse	1	2	3	4	5
g.	Boiterie persistante ou nouvelle	1	2	3	4	5
h.	Différence de longueur	1	2	3	4	5
i.	Incapacité de reprendre toutes les activités	1	2	3	4	5
j.	Infection de la prothèse	1	2	3	4	5
k.	Déboitement de la prothèse (luxation)	1	2	3	4	5
l.	Fracture de l'os entourant la prothèse	1	2	3	4	5
m.	Fracture de la prothèse elle-même	1	2	3	4	5
n.	Perte du contrôle de sa santé	1	2	3	4	5
o.	Effets secondaires ou maladies causés par les débris ou les particules de métal ou de plastique qui se répandent dans le corps	1	2	3	4	5
p.	Mauvaise acceptation de la prothèse (corps étranger) par le corps	1	2	3	4	5
q.	Changement précoce de la prothèse pour cause d'usure ou de descellement (=durée de vie courte)	1	2	3	4	5

k. Autre(s) problème(s), veuillez préciser :

....

....

....

Dans la liste ci-dessus (a à k), merci d'indiquer les trois conséquences indésirables <u>les plus importantes</u> à vos yeux, par ordre d'importance :

(Merci de mentionner la lettre correspondante, par exemple « a » pour risque de saignement important, etc.)

1. ___________ 2. _____________ 3. ______________
