# Supplementary material for: Development of a patient-centred tool for use in total hip arthroplasty
Source: PLoS One. 2024 Oct 24;19(10):e0307752. doi: 10.1371/journal.pone.0307752 (PMC11500863; doi:10.1371/journal.pone.0307752)
Supplement: S2 File — (PDF) [file pone.0307752.s002.pdf]

# Bénéfices et risques de la prothèse totale de hanche - Questionnaire patient-e

1. Concernant la pose d'une prothèse de la hanche, dans quelle mesure les **<u>bénéfices</u>** suivants sont, pour vous, importants ou pas ?

   *Merci de répondre par rapport à la(les) prothèse(s) que vous avez eue(s), ou que vous allez avoir.*

*Pour chaque ligne ci-dessous, veuillez entourer le chiffre approprié :*

| | Pas du tout important | | | | Très important | Ne s'applique pas à moi |
|---|---|---|---|---|---|---|
| a. Disparition de la douleur | 1 | 2 | 3 | 4 | 5 | |
| b. Arrêt ou diminution des médicaments contre la douleur | 1 | 2 | 3 | 4 | 5 | |
| c. Retrouver le sommeil | 1 | 2 | 3 | 4 | 5 | |
| d. Bien-être émotionnel | 1 | 2 | 3 | 4 | 5 | |
| e. Indépendance dans la marche et les déplacements | 1 | 2 | 3 | 4 | 5 | |
| f. Reprise de mes activités quotidiennes à la maison | 1 | 2 | 3 | 4 | 5 | |
| g. Reprise de mes activités professionnelles | 1 | 2 | 3 | 4 | 5 | ❑ |
| h. Reprise de mes activités de loisirs (sport, voyages, etc.) | 1 | 2 | 3 | 4 | 5 | |
| i. Reprise de ma vie sociale (voir ses amis, voir sa famille) | 1 | 2 | 3 | 4 | 5 | |

j. Autre(s) bénéfice(s) pour moi, veuillez préciser :..........................................................................................

..........................................................................................................................................................

..........................................................................................................................................................

*Parmi la liste ci-dessus (a à j), merci d'indiquer les trois bénéfices <u>les plus importants</u> à vos yeux, par ordre d'importance :*
*(Merci de mentionner la lettre correspondante, par exemple « a » pour la disparition de la douleur, etc.)*

1. __________ 2. _____________ 3. _____________

2. Pour certaines personnes, bénéficier d'une prothèse de hanche est synonyme de retrouver son autonomie, son indépendance, de récupérer la pleine possession de ses moyens. Dans quelle mesure êtes-vous d'accord avec cette opinion ?

   *Merci d'entourer le chiffre approprié ci-dessous*

| Pas du tout d'accord | | | | Tout à fait d'accord |
|---|---|---|---|---|
| 1 | 2 | 3 | 4 | 5 |

3. Concernant la pose d'une prothèse de la hanche, dans quelle mesure les **<u>conséquences indésirables ou les problèmes</u>** suivants sont, pour vous, <u>importants</u> ou pas ?

*Merci de répondre par rapport à la(les) prothèse(s) que vous avez eue(s), ou que vous allez avoir.*

*Pour chaque ligne ci-dessous, veuillez entourer le chiffre approprié :*

| | | Pas du tout important | | | | Très important |
|---|---|---|---|---|---|---|
| a. | Saignement important | 1 | 2 | 3 | 4 | 5 |
| b. | Caillot de sang (thrombose veineuse profonde) | 1 | 2 | 3 | 4 | 5 |
| c. | Décès lors de l'opération | 1 | 2 | 3 | 4 | 5 |
| d. | Douleur persistante | 1 | 2 | 3 | 4 | 5 |
| e. | Douleur qui se répand sur d'autres articulations | 1 | 2 | 3 | 4 | 5 |
| f. | Un bruit persistant de ma prothèse | 1 | 2 | 3 | 4 | 5 |
| g. | Boiterie persistante ou nouvelle | 1 | 2 | 3 | 4 | 5 |
| h. | Différence de longueur | 1 | 2 | 3 | 4 | 5 |
| i. | Incapacité de reprendre toutes mes activités | 1 | 2 | 3 | 4 | 5 |
| j. | Infection de ma prothèse | 1 | 2 | 3 | 4 | 5 |
| k. | Déboitement de ma prothèse (luxation) | 1 | 2 | 3 | 4 | 5 |
| l. | Fracture de l'os entourant ma prothèse | 1 | 2 | 3 | 4 | 5 |
| m. | Fracture de ma prothèse elle-même | 1 | 2 | 3 | 4 | 5 |
| n. | Perte du contrôle de ma santé | 1 | 2 | 3 | 4 | 5 |
| o. | Effets secondaires ou maladies causés par les débris ou les particules de métal ou de plastique qui se répandent dans mon corps | 1 | 2 | 3 | 4 | 5 |
| p. | Mauvaise acceptation de ma prothèse (corps étranger) par mon corps | 1 | 2 | 3 | 4 | 5 |
| q. | Changement précoce de ma prothèse pour cause d'usure ou de descellement (=durée de vie courte) | 1 | 2 | 3 | 4 | 5 |

k. Autre(s) problème(s) pour moi, veuillez préciser : ..........................................................................................

..............................................................................................................................................................

..............................................................................................................................................................

*Dans la liste ci-dessus (a à k), merci d'indiquer les trois conséquences indésirables <u>les plus importantes</u> à vos yeux, par ordre d'importance :*

*(Merci de mentionner la lettre correspondante, par exemple « a » pour risque de saignement important, etc.)*

1. ___________ 2. ______________ 3. ______________
